# Potential Molecular Signatures Predictive of Lung Cancer Brain Metastasis

**DOI:** 10.3389/fonc.2018.00159

**Published:** 2018-05-11

**Authors:** Rute M. S. M. Pedrosa, Dana A. M. Mustafa, Joachim G. J. V. Aerts, Johan M. Kros

**Affiliations:** ^1^Department of Pathology, Erasmus Medical Center, Rotterdam, Netherlands; ^2^Department of Lung Diseases, Erasmus Medical Center, Rotterdam, Netherlands

**Keywords:** brain metastasis, lung cancer, molecular mechanisms, genetic alterations, chemotherapy

## Abstract

Brain metastases are the most common tumors of the central nervous system (CNS). Incidence rates vary according to primary tumor origin, whereas the majority of the cerebral metastases arise from primary tumors in the lung (40–50%). Brain metastases from lung cancer can occur concurrently or within months after lung cancer diagnosis. Survival rates after lung cancer brain metastasis diagnosis remain poor, to an utmost of 10 months. Therefore, prevention of brain metastasis is a critical concern in order to improve survival among cancer patients. Although several studies have been made in order to disclose the genetic and molecular mechanisms associated with CNS metastasis, the precise mechanisms that govern the CNS metastasis from lung cancer are yet to be clarified. The ability to forecast, which patients have a higher risk of brain metastasis occurrence, would aid cancer management approaches to diminish or prevent the development of brain metastasis and improve the clinical outcome for such patients. In this work, we revise genetic and molecular targets suitable for prediction of lung cancer CNS disease.

## Introduction

Brain metastases are the most common tumors of the central nervous system (CNS). Metastatic brain lesions outnumber primary brain tumors with a 10-fold (1) with incidence rates varying according to the primary tumor origin. The majority of the cerebral metastases arise from primary tumors in the lung (40–50%) and it is estimated that 50% of the patients with small-cell lung cancer (SCLC) or non-small-cell lung cancer (NSCLC) will develop brain metastasis (2, 3). In contrast to cerebral metastases from other primary cancers, where generally a metastatic latency period takes place, brain metastasis from lung cancers often occur months after, or even concurrently, with the diagnosis of the primary tumor (4). Metastatic brain lesions carry a clinical burden of morbidity and mortality, as well as significant neurological deficits, cognitive impairment, and emotional difficulties (5). Despite treatment, lung cancer brain metastases are usually fatal for 90% of patients within two years after the initial diagnosis, with a median survival of 7–10 months five years after diagnosis (2). Previous efforts to characterize patients that are at high risk of developing brain metastasis have been fairly disappointing.

Currently, only clinical and pathologic variables are used to predict the risk of brain metastasis in patients with lung cancer. However, data on predictive parameters are diverse and not clinically usable (Table 1). Identifying patients at highest risk of developing brain metastases on the basis of standard clinical and pathological factors, such as status of primary tumor, tumor histology, nodal involvement, and patient age, may not be reliable due to small hazard ratios and unknown prognostic factors (6). Recently, Hung et al. (7) demonstrated in a study on 182 lung adenocarcinomas with distant metastases that the micropapillary histology subtype was significantly associated with brain metastasis (*p* = 0.01). However, a more robust method to identify which patients are at risk of developing brain metastasis is urgently needed.

**Table 1:** Conflicting clinical and pathological risk factors associated with the development of brain metastases.

Reference	Analysis	*N* =	Type Tumor	Pathologic stage	Recurrence site	Age	Tumor status	Lymph-vascular space invasion	Nodal status	Histologic type
Ceresoli et al. (8)	Multivariate	112	Non-small-cell lung cancer (NSCLC)	IIB–IIIB	Brain	<60, *p* = 0.03	ND	ND	*p* = 0.003*	Non-squamous+
Andre et al. (9)	Multivariate	267	NSCLC	IIIN2	Brain	ND	−	ND	ND	Adenocarcinoma+
Bajard et al. (10)	Multivariate	305	NSCLC	I–IIIB	Brain	<62, *p* = 0.004	T4, *p* = 0.0009	ND	N2-3, *p* = 0.0057	Adenocarcinoma, *p* = 0.0002
Carolan et al. (11)	Multivariate	83	NSCLC	IIIB	Brain	<60, *p* = 0.022	ND	ND	ND	−
Chen et al. (12)	Kaplan-Meier	211	NSCLC	IIIA	Brain	−	−	ND	ND	Squamous vs non-squamous, *p* = 0.02
Hubbs et al. (3)	Multivariate	975	NSCLC	I–II	Brain	<77, *p*<0.01	Size, *p* < 0.01	*p* = 0.03	*p* = 0.04	−
Jacobs et al. (13)	Multivariate	78	NSCLC	II, III	Brain	−	−	ND	N1-2 vs N0, *p* < 0.02	ND
Mujoomdar et al. (14)	Hierarchical logistic regression	264	NSCLC	I–IV	Brain	−	Size, *p* < 0.001	ND	*p* < 0.017	Adenocarcinoma+ undifferentiated vs squamous, *p* = 0.001
Robnett et al. (15)	Multivariate	150	NSCLC	II, III	Brain	−	ND	ND	ND	IIIB non-squamous+
Schouten et al. (16)	Univariate	2724	Div.	I–IV	Brain	<70 (breast and lung cancer)	ND	ND	ND	ND
Tang et al. (17)	Univariate	25	NSCLC	I–III	Brain	−	−	ND	Mediastinale vs hilar, *p* = 0.03	ND
Tang et al. (18)	Multivariate	292	NSCLC	ND	Brain	−	T2 vs T3–4, *p* = 0.005*	ND	N0–1 vs N2–3, *p* < 0.001*	−
Tsuchiya et al. (19)	Multivariate	322	NSCLC	IA	Brain and others	−	Size ≥15 mm, *p* = 0.038	ND	ND	Squamous, *p* = 0.002
Westeel et al. (20)	Multivariate	192	NSCLC	I–IV	Brain and others	<61, *p* = 0.01	ND	ND	ND	ND

*ND, not determined; “−”, no predictive value (*p* > 0.05); “+”, predictive value, no significance*.

*Significant for univariate analysis only.

Molecular classification by correlating distinct molecular markers with oncogenic mechanisms has been practiced to improve risk stratification of the TNM staging system (21). The potential of molecular biologic distinction would direct appropriate therapy, thereby improving patient outcome. Among early-stage (I/II) NSCLC patients, the 5-year overall survival (OS) rate is only 45.1% (22). Many clinical trials have confirmed that postoperative adjuvant therapy can prolong the survival of NSCLC patients. In a recent meta-analysis of 3,923 patients, Chen et al. (23) demonstrated the efficacy of postoperative chemotherapy – both cisplatin based (*p* < 0.0001) and single tegafur-uracil (UFT, *p* = 0.002), in stages I–II, IA, and IB NSCLC, and no significant benefit was found in stage IA patients (*p* = 0.43). In addition, cisplatin was shown to be better than single UFT chemotherapy in OS (*p* = 0.0005 and *p* = 0.81, respectively) (23). More trials should be conducted in order to confirm the efficacy of disease-free survival therapies in future clinical practice.

In order to predict the rise of cerebral metastasis of lung cancer, we would need a measurable biomarker that correlates well with brain seeding of the lung cancer cells. Molecular markers may be classified into subgroups based on their mechanism of action in the metastatic cascade to the brain (6). The optimal marker to disclose concealed (brain) metastatic disease would be displayed in primary tumors while not detectable in the serum of control subjects (24). The capacity to identify metastatic disease based on proto-oncogenes such as Kirsten rat sarcoma viral oncogene homolog (KRAS) and tumor suppressor p53 (TP53), present in only half of the lung cancer patients (47 and 50%, respectively) (25), demands a more broaden and deepened spectrum of the investigation of primary lung cancers, the molecular interactions with other cells, and the tumor microenvironment.

The process of metastasis is a selective and refined event called organotropism whereby, apart from an overall tendency to spread and invade, primary tumors show predilection for particular distinct organs (26). Cancers that metastasize to brain need to take a number of anatomic, physiologic, and molecular hurdles. The first requirement is intravasation into the blood stream, dependent upon a reversible epithelial-to-mesenchymal transition (EMT). The epithelial cell traits, such as cell polarity and E-cadherin-mediated cell adhesion, are suppressed and replaced by mesenchymal cell characteristics. The cells become motile, invasive, and resistant to apoptosis (27). Through the EMT process, tumor cells acquire stem cell-like features such as self-renewal, differentiation and ability to seed, justifying the term “tumor-initiating cells” (27). EMT molecular regulation is accomplished through an intricate network arranged by different genes and molecule inducers of EMT (28–30). AXL, a receptor tyrosine kinase belonging to the TAM family, and its ligand GAS6, growth arrest-specific gene 6, have been reported to down-regulate several oncogenic signaling pathways (31), through activation of MAPK/ERK and PI3K/AKT signaling pathways (32, 33). Recently, AXL-GAS6 signal axis has been reported to have a potential key role in NSCLC tumor progression and may be suitable as a prognostic biomarker for identifying high-risk NSCLC brain metastasis patients (34). Tumor cell growth in the brain microenvironment is the result of genetic predisposition and cellular adaptation mechanisms and is largely dependent on cross-talk between tumor and brain-resident cells.

Genomic instability and mutations are just two of the characteristics of cells associated with the transition from a preneoplastic lesion to an invasive tumor state and consequent progression to metastatic disease. During tumorigenesis, a sequence of genetic modifications such as gene deletions, copy number alterations (CNAs), and chromosomal rearrangements occur. This review focuses on the use of molecular characteristics that are predictive of tumor progression and development of metastatic NSCLC brain metastasis in particular.

## Genetic Alterations

**Figure 1:**
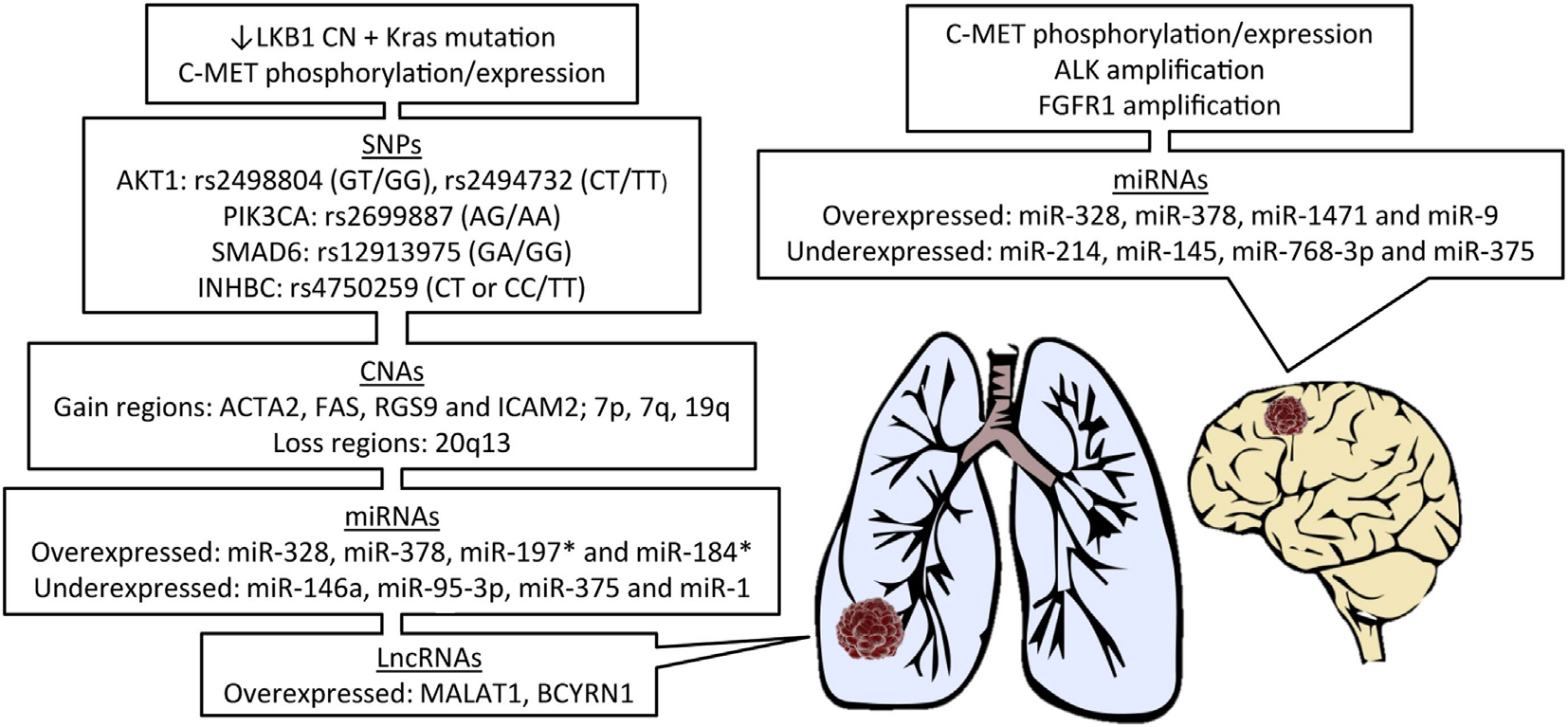
Features of primary lung cancer from patients known to develop brain metastasis and potential biomarker candidates. LKB1, liver kinase B1; CN, copy number; SNPs, single-nucleotide polymorphisms; CNAs, CN alterations; miRNAs, microRNAs; lncRNAs, long non-coding RNAs; *in EGFR-mutant patients.

Due to the recent discoveries of targetable genetic alterations in the treatment of NSCLC, patients have been stratified according to genetic variations in the primary tumor, including epidermal growth factor receptor (EGFR), KRAS, and anaplastic lymphoma kinase (ALK) (35). A summary of all genetic alterations that will be addressed in this review are presented in Figure 1. There are considerable differences in reported incidence and time to development of brain metastases for these genetic alterations.

## Epidermal Growth Factor Receptor

In the Caucasian population, EGFR-activating mutations are present in 10–15% of adenocarcinomas and in less than 5% of squamous cell carcinomas (36). Roughly 90% of all known EGFR mutations reside in exon 19 (in-frame deletions) and in exon 21 (L858R, point mutation) (37, 38). The prevalence of activating EGFR mutations appears to be dependent on gender, smoking status and ethnicity. In patients from East-Asia, EGFR mutation is reportedly up to five times higher than in Caucasian patients (39–41). The relation between EGFR status, brain metastasis and survival are complex and not fully understood. It has been shown that lung cancer patients suffering from tumors with particular EGFR mutations survive longer, probably due to effective treatment. However, data also suggest that brain metastases arise more frequently in patients with primary lung tumors bearing EGFR mutations (42, 43) and the development of brain metastases is relatively frequent during treatment. There are discordance rates of EGFR mutational status between primary tumors and their CNS metastases that vary from 0 to 32% (44–50). In a series of 55 NSCLC primary tumors with matched cerebral metastases, EGFR was found to be more frequently amplified in the metastatic adenocarcinomas than corresponding primary tumors, with 30 and 10%, respectively (50). Discrepancies regarding the response of the brain metastases may well be due to the timing of administering adjuvant chemotherapy for the primary tumors relative to the occurrence of the brain metastases. The choice of the agents is currently based on the molecular characteristics of the primary, not the metastatic, tumors. Paradoxically, prolonging survival times due to successful response of the primary tumors would create more time for brain metastases to develop as late complication (51, 52). Similar to EGFR, the KRAS status may also be discordant between primary and metastatic tissues (44) and a KRAS mutation in a small subset of tumor cells may confer resistance to EGFR tyrosine kinase inhibitors (TKIs) therapy.

## KRAS

Epidermal growth factor receptor and KRAS mutations are generally mutually exclusive (53, 54), but cases of EGFR and KRAS co-mutations have been identified (55–57). Roughly 15–30% of NSCLCs harbor activating mutations in codons 12 and 13 of the KRAS gene (58). KRAS mutations are associated with advanced tumor progression and clinical aggressiveness (59), forming a persistent risk of lung adenocarcinoma and implying to be an early event in the tumorigenesis process (53). The correlation of the presence of KRAS mutations with a smoking history (60) suggests that KRAS mutations are a sequel of the actions of carcinogens of tobacco products (53). However, in a cohort of 482 lung adenocarcinomas, it was demonstrated that KRAS mutations do occur in patients with lung adenocarcinomas without a smoking history (61), but the mutations are different. Significantly more transition mutations (G>A) are being found in non-smokers than the transversion mutations (G>T or G>C, *p* < 0.0001) that occur in former- or current smokers (61). This observation supports the idea that the distinct transition profile – replacement of a purine for a purine or a pyrimidine for a pyrimidine (62) – of never smokers is very unlikely to be caused by passive tobacco vulnerability. No specific KRAS targeting treatment has so far shown efficacy. There is little available data on the KRAS mutational status in primary lung cancers as compared to that in their brain metastases (44, 57). In a relatively small series, Munfus-McCray et al. found 23.5% of brain metastatic lung adenocarcinomas with KRAS mutation exclusively in patients with a smoking history (*p* < 0.01) (59).

## Anaplastic Lymphoma Kinase

Anaplastic lymphoma kinase rearrangements occur in 2–7% of all NSCLC, with predominance in non- or light smokers, younger age, and adenocarcinomas (63, 64). Fusion between EML4 (echinoderm microtubule-associated protein-like 4) and ALK yields at least 15 molecular variants with different biological behaviors and affected signaling pathways and consequences for therapy choice (65). ALK testing is particularly recommended for non-squamous lung cancers in the absence of EGFR mutation, of patients with non- or light smoking history (66). The recommended method for testing the presence of ALK translocation is fluorescent *in situ* hybridization (FISH) and immunohistochemistry (IHC) as confirmation (67, 68). In a large Western cohort, functional ALK rearrangements appeared to be mutually exclusive with EGFR and KRAS mutations (69). Although ALK translocations seem to be similar in primary tumors and their brain metastases, ALK amplifications are found more frequently in CNS metastasis with discordance rates of only 12.5% (70). Similar to EGFR, ALK rearrangements are predictive of response to TKIs, but the development of brain metastasis in patients with ALK translocations receiving ALK directed TKI is a major clinical problem (71, 72). Recently, second-generation TKI alectinib has shown to delay the development of brain metastases compared to first-generation TKI and also demonstrated promising efficacy in the CNS for crizotinib-resistant ALK-positive NSCLC patients (73, 74). Similar to EGFR and KRAS mutations and ALK rearrangements, several other molecules such as liver kinase B1 (LKB1, also known as STK11), proto-oncogene tyrosine-protein kinase ROS1, and C-MET that encodes the hepatocyte growth factor receptor were found to be implicated in the development of lung cancer (75–79). However, the connection of these molecules with the development of brain metastases is still under investigation and not yet implicated in clinical decision making. KRAS aberrations have a synergistic effect with LKB1 inactivation on lung cancer development and distant metastasis formation (80, 81). In a cohort of 154 NSCLC patients, Zhao et al. demonstrated that a lower LKB1 copy number (CN), along with KRAS mutation, were significantly associated with a higher number of brain metastasis. Moreover, the odds ratio of brain metastasis was ~20 times higher in patients with one decrease in LKB1 CN values (82). LKB1 is observed to be inactivated in ~30% of all NSCLCs (83).

## Other Mutations

Although several potential targets may not regularly be expressed in a high number of lung cancer brain metastasis, their potential use for personalized treatment of selected lung cancer patients harboring actionable mutations should not be discarded. In a cohort of 874 brain metastases samples, of which 295 NSCLC, Capper et al. showed that, although a total of 51/874 samples harbored a BRAF V600E mutation, only 1/295 NSCLC brain metastases (~0.3%) was BRAF mutant (84). Despite this low frequency of BRAF mutations in lung brain metastasis, regression of both visceral and brain metastases by BRAF inhibitor vemurafenib was reported in a patient with a BRAF V600E-mutated NSCLC (85). While 3% of primary lung cancers harbor ROS1 alterations, only 1/99 adenocarcinomas bore ROS1 translocations and 1/11 squamous cell carcinomas showed ROS1 amplifications (86).

Activating mutations in EGFR are associated with sensitivity to TKI therapy, but nearly 30% of EGFR positive patients show primary resistance to EGFR inhibitor therapy (87). While C-MET amplification is one of the factors commonly associated with disease progression (88), Benedettini et al., in a first cohort of 23 NSCLC samples of patients harboring an EGFR activating mutation, showed that both C-MET phosphorylation and expression were significantly associated with shorter time to progression, correlating with *de novo* resistance to EGFR TKI. In a second cohort of 40 patients, englobing 18 primary NSCLC from patients who later developed brain metastases and 22 NSCLC from patients that did not develop brain metastases, Benedettini et al. demonstrated that both C-MET expression and phosphorylation, but not C-MET amplification, were significantly higher in the tumors from patients who developed brain metastasis. In 18 matched brain metastasis, amplification was demonstrated (89). In addition, in a cohort of 196 NSCLC brain metastasis samples, Presseur et al. found C-MET gene amplification and overexpression in 21.6 and 44.4%, respectively, confirming that C-MET is commonly activated in brain metastasis manifestation (90). Furthermore, a significant correlation between C-MET and ALK amplification status was observed (*p* = 0.039). In another study, these authors demonstrated that fibroblast growth factor receptor 1 (FGFR1) amplification in brain metastases of adenocarcinomas – but not squamous cell carcinomas, is fivefold more frequent than reported for primary tumors (~3%). Similar to C-MET, a positive correlation of ALK and FGFR1 amplification status in brain metastasis was reported as significant (*p* < 0.001) (91). In a recent study, Keap1, Nrf2, and P300, key genes of the Keap1–Nrf2–ARE survival pathway, were found to be mutated in brain metastatic tissue of progressive NSCLC patients (92). Moreover, mutations in Keap1-Nrf2-ARE pathway were found in circulating tumor cells (CTCs), suggesting a role in the ability of CTCs to bear the rough environment in blood-circulation and attain distant organs (92).

## Circulating Tumor DNA (ctDNA)

An adequate characterization of somatic genetic modifications in human cancers is critical for an optimal diagnosis and subsequent therapy. In brain metastatic tissue, as for all other brain malignancies, repeated biopsies are not a feasible approach to portray the tumor clonal diversity. Several studies have shown that cell-free ctDNA in the plasma could serve to characterize and monitor tumors (93–95). Nevertheless, ctDNA analysis of plasma from patients with brain malignancies has disclosed very low levels of tumor DNA (96). Recently, ctDNA analysis from cerebrospinal fluid (CSF) has been shown promising for brain cancer patients (97–99) and brain metastatic cancer patients (100, 101). CSF is in direct contact with the brain and, therefore, with tumor cells of brain cancer patients. In a comparative study of ctDNA derived from plasma and from CSF of patients with primary or metastatic brain tumors, De Mattos-Arruda et al. showed ctDNA levels of brain malignancies to be more abundantly present in the CSF than in the plasma (100). Moreover, ctDNA from CSF appeared to recapitulate the brain metastasis-specific mutations – private mutations, absent in extracranial tumors of a patient with Her2-positive metastatic breast cancer (100). In addition, the CSF ctDNA proficiency to monitor responses to systemic therapy and brain tumor progression (98, 100), i.e., the capacity of the CSF ctDNA to recapitulate the modulation of mutant allele frequency over time in the brain tumor burden, suggests that genomic CSF analysis may be useful not only in facilitating diagnosis of tumor in the CNS or as guidance to second-line agents choice, but also in pinpointing pathways’ intimate related with cancer spread to the CNS and predictive of brain metastases (98).

## Single-Nucleotide Polymorphisms (SNPs) Associated with Brain Metastases

Studying SNPs in signaling pathways that regulate cell proliferation and migration and assessing the relationship between multiple SNPs can be used to estimate the risk of brain metastasis. The PI3K–PTEN–AKT–mTOR pathway, important in the control of cell growth, tumorigenesis, and cell invasion, has been shown to be abnormally activated in several cancer types, including NSCLC (102, 103). In a study of genetic variations in the PI3K–AKT–mTOR pathway to predict brain metastasis in NSCLC patients, Quianxia et al. identified three SNPs that appeared to be exclusively associated with higher risk of brain metastasis: the GT/GG (*p* = 0.006) and CT/TT (*p* = 0.002) genotypes of AKT1, variant alleles rs2498804 and rs2494732, respectively, and AG/AA (*p* = 0.010) genotype of PIK3CA, variant allele rs2699887 (103). Furthermore, patients carrying at least one variant allele in PIK3CA had roughly twice the risk of brain metastasis as those without those variants (103). Multiple mechanisms of PI3K activation may be responsible for activation of the PI3K pathway (104), and increased PI3K activity would result in increased metastases. In concordance, Paik et al. reported that patients with aberrant PI3K squamous lung carcinomas (*n* = 9) had worse survival (median OS: 8.6 vs 19.1 months, *p* < 0.001), higher metastatic burden (>3 organs, 18 vs 3%, *p* = 0.025), and higher incidence of brain metastases (27 vs 0%, *p* < 0.001) (105). Similar to PIK3K–AKT–mTOR pathway, it was hypothesized that common genetic variants in the TGF-β pathway would be associated with the risk of brain metastasis (106). TGF-β pathway has been demonstrated to suppress early-stage tumor development and to stimulate tumor cell growth and invasiveness at later stages of tumorigenesis (107). Quianxia et al. found the GG genotype of SMAD6: rs12913975 (*p* = 0.014) and the TT genotype of INHBC: rs4750259 (*p* = 0.024) to be associated with risk of brain metastasis in a cohort of 161 blood samples from NSCLC patients. Furthermore, de combination of both genetic variants was shown to be higher for prediction of brain metastasis (*p* = 0.001) (106).

## CNAs Associated with Brain Metastasis

Activation or inhibition of a gene occurs through a variety of mechanisms such as, for example, activating mutations and deletions. Gene deletion can be evaluated by CNA. Animal models have given clear evidence that LKB1 haploinsufficiency stimulates KRAS driven lung cancer in mice (81), and a single copy inactivation of LKB1 can considerably ease brain recurrence (82). Although EGFR CN status is still controversial and some of the available data do not support EGFR CN as a prognostic factor (108, 109), Bonanno et al. have shown, despite the less predictive accuracy of FISH analysis compared to EGFR mutation analysis, that patients with EGFR-FISH-positive tumors have better outcomes (median OS: 177 vs 57 weeks, *p* = 0.048) (110). Considering that primary lung adenocarcinomas with early development of brain metastasis would contain more CNAs predictive of metastatic potential, Lee et al. compared the CN changes of four lung adenocarcinomas with coexistent brain metastasis with 8 lung adenocarcinomas with metachronous brain metastasis (111). Amplification in 5q35.1-2 and 17q23.3-24.1 was detected in 100% and that in 10q23.31 and 17q24.1 was detected in 75% of the cases with synchronous brain metastasis. On the other hand, and in a less frequent ratio, only 5q35.1-2 and 17q24.1 amplification status was found in 12.5% of the metachronous brain metastasis. Moreover, gained regions specific for early (simultaneous) brain metastasis were found to contain ACTA2, FAS, RGS9, and ICAM2 as putative metastasis promoting genes, the latter being most significant (*p* = 0.002) (111). In the same line, another study compared CNAs of primary NSCLC tumor and matched brain metastasis from one single patient (112). Brain metastatic tissue exhibited a higher degree of genetic heterogeneity when compared with the primary tumor with common regions of gain including 7p, 7q, and 19q and common regions of loss including 20q13 (112). In a stage IV SQCLCs study, four brain metastases and matched archived FFPE primary cancers were shown to have complete loss of PTEN by IHC and whole exome sequencing (105). In an early-stage NSCLC report, 30 (24%) of the total of 125 specimens analyzed for PTEN-IHC showed a lack of staining (113). Although genetic alterations of the PTEN gene are unusual in NSCLC, loss of PTEN protein is not a unique event in early-stage NSCLC and Soria et al. demonstrated that besides being a reversible event, PTEN loss may be partially explained by promotor methylation, in addition to point mutations and homozygous deletions (113).

## Micrornas (miRNAs) Associated with Brain Metastasis

Recently, molecular studies have stressed the role of miRNAs which are small non-coding endogenous RNAs containing 18–24 nucleotides that regulate gene expression at the post-transcriptional level thereby acting as negative regulators of mRNA translation and/or stability (114). miRNAs appear to regulate several hundred genes and could serve as a better classifier than gene expression profiling (115). miRNAs are known to play a crucial role in normal development, proliferation, differentiation, and apoptosis, and dysregulation of miRNAs has been linked to various pathological conditions, including cancer (116). The role of miRNAs in the development of brain metastases has been recently explored (117, 118).

Several studies have addressed the miRNA expression as biomarkers to predict the occurrence of brain metastases in lung cancer. miRNA-328 appeared to be significantly overexpressed in both primary tumor samples and cerebral metastases of patients with NSCLC, when compared with NSCLC patients without brain metastasis. Moreover, miRNA-328 overexpression has been found to promote migration and subsequent brain metastasis formation of NSCLC cells through PRKCA deregulation (119). PRKCA mediates the expression of urokinase plasminogen activator, leading to the migration of the tumor cells (120). Similar to miRNA-328, miRNA-378 has also been demonstrated as a potential biomarker to assist clinicians in stratifying patients for high-risk of brain metastasis, because miRNA-378 was also found to be overexpressed in NSCLC primary tumor samples and matched brain metastasis of NSCLC patients (121). Also, miRNA-378 promotes cell migration, invasion, tumor growth, and angiogenesis, *in vitro* and *in vivo* (121). Recently, Remon et al. have identified miRNA-197 and miRNA-184 as two significantly overexpressed miRNAs in EGFR-mutant patients with brain metastases, when compared with EGFR-mutant patients with no brain metastasis (122). However, because of lack of patients with EGFR wild-type (EGFRwt) tumors without BM, no comparison between patients with EGFRwt tumors, with and without BM, could be made. Therefore, the effects of these miRNAs, irrespective of the EGFR status, need further scrutiny.

MicroRNAs’ expression status varies according to their targeted genes. Zhao et al. have reported the significant up-regulation of miRNA-1471 and miRNA-9 and down-regulation of miRNA-214 and miRNA-145 in 11 brain metastatic lung cancer samples, when compared with 40 primary lung adenocarcinomas (*p* < 0.001 for all four miRNAs) (123). The up-regulation of miRNA-145 in primary lung adenocarcinomas was shown to suppress proliferation of tumor cells (123), consistent with other reports that show inhibition of cell proliferation in human lung adenocarcinomas through miRNA-145 targeting c-Myc, EGFR and NUDT1 (124, 125). Subramani et al. have shown the miRNA-768-3p to be underexpressed in several brain metastases, compared to matched primary tumors (126). miRNA-768-3p was found to be underexpressed in *in vitro* lung cancer cells after co-culture with astrocytes, driving to increased KRAS protein and downstream effectors ERK1/2 and BRAF, thereby boosting tumor cell viability and promoting metastasis. From various studies, it appears that miRNAs regulate the growth of metastases either by under- or overexpression, within the tumor tissue or in the tumor environment. The brain microenvironment negatively regulates miRNA-768-3p to enhance KRAS expression that promotes the propagation of lung cancer brain metastasis (126). miRNA-146 was shown to be significantly up-regulated in NSCLC tissue when compared to healthy adjacent lung tissue (*p* < 0.05) (127). In another study, miRNA-146a expression in primary NSCLC was correlated with advanced clinical TNM stages and distant metastasis (*p* < 0.05). The patients with a high miRNA-146a expression showed longer progression-free-survival times than those with a low expression of miRNA-146a (25.6 and 4.8 weeks, respectively, *p* < 0.05) (128). In the same line with these findings, in a xenograft model, Hwang et al. showed high expression of miRNA-146a in parental cells, while diminished expression in the brain-seeking cells. Moreover, miRNA-146a overexpression in the brain-seeking cancer cells suppressed their metastatic potential, which was correlated to the up-regulation of β-catenin and down-regulation of heterogeneous nuclear ribonucleoprotein C1/C2 (129). Taken together, these findings suggest that miRNA-146a serve as a valid clinical biomarker for prediction of brain metastasis in lung cancer patients. However, validation of miRNA-146a expression levels in a large cohort of human matched primary and brain metastatic lung tumors is essential to confirm this finding. Similar to miRNA-146a, overexpression of miRNA-95-3p suppresses brain metastasis of lung adenocarcinoma through down-regulation of cyclin D1 (130). miRNA-95-3p is decreased in brain metastases of lung cancers as compared to the primary tumors and higher cyclin D1 expression correlates with poorer prognoses (130). In a recent study, Chen et al. reported miRNA-375 deregulation to be associated with NSCLC brain metastasis (131). miRNA-375 is another miRNA documented to be down-regulated in primary tumors of NSCLC patients with brain metastasis. miRNA-375 expression was significantly decreased in matched brain metastatic NSCLC tissues (*p* < 0.05) and significantly correlates with total number of brain metastasis (*p* < 0.001). In addition, VEGF and MMP9 – which roles have been extensively studied in the development of brain metastasis – were over-expressed in down-regulated miRNA-375 tumors (131).

MicroRNAs are linked with several molecular pathways. Several studies have correlated the overexpression of ADAM9 in NSCLC patients with brain metastases (4, 132). ADAM9 has been demonstrated to enhance the ability of tissue plasminogen activator to cleave and stimulate the function of CUB domain containing protein 1 (CDCP1) – promigratory protein, to promote brain metastasis (4). Recently, Chiu et al. reported that ADAM9 down-regulates miRNA-1 via EGFR signaling pathways activation, enhancing CDCP1 expression to promote lung cancer progression (133). miRNA-1 expression was shown to be down-regulated in primary lung tumors but increased in ADAM9-knockdown lung cancer cells. Moreover, miRNA-1 negatively correlates with CDCP1 expression and with migration ability of lung cancer cells (133). Another study has identified miRNA-21 as a target of signal transducers and activators of transcription 3 (STAT3) pathway activity in lung-derived brain metastasis initiating cells (134). STAT3 is admitted as a central regulator in the metastatic process (135), and STAT3-knockdown has been demonstrated to reduce expression of known downstream targets of miRNA-21, while STAT3 and miRNA-21 act as cooperative regulators of stemness, migration and tumor initiation in lung-derived brain metastasis (134). miRNAs appear very promising as diagnostics, prognostics and therapeutics to improve cancer patient outcome; however, the clinical use of miRNA therapeutics to treat brain metastases has yet to be achieved. Advances in pre-clinical and translational studies to identify miRNAs that change after growth in the brain microenvironment have been made, but validation of large cohorts from patient tumor samples is required.

## Long Non-Coding RNAs (lncRNAs) Associated with Brain Metastasis

Long non-coding RNAs have been recently identified as effective players in tumorigenesis. lncRNAs represent a class of non-protein coding transcripts longer than 200 nucleotides (136) that covers a broad spectrum of physiological and pathological functions by implementing different modes of action (137). Similar to miRNAs that regulate several hundred genes, lncRNAs are involved in the regulation of multiple miRNAs, impacting the expression of thousands of genes (136). Besides performing a single function, some lncRNAs act at multiple functional levels in different types of cells. Metastasis-associated lung adenocarcinoma transcript 1 (MALAT1), localized in nuclear speckles and highly conserved among mammals, regulates alternative splicing (138) and gene expression through additional splicing-independent mechanisms in lung cancer metastasis (139). In a recent study, Shen et al. have shown lncRNA-MALAT1 levels to be significantly higher in primary NSCLC from patients who developed brain metastasis when compared with primary NSCLC from patients without brain metastasis (*p* < 0.001) (140). Additional *in vitro* functional studies showed overexpression of vimentin in a highly invasive subline of brain metastasis lung cancer cells overexpressing MALAT1, while overexpression of E-cadherin was observed when MALAT1 was silenced, indicating that MALAT1 overexpression promotes lung cancer brain metastasis by inducing EMT (140). Accordingly, RNAi-mediated suppression of MALAT1-RNA, negatively influenced migration and clonogenic growth in established human NSCLC cell lines. Forced expression of MALAT1 in mouse NIH 3T3 fibroblasts significantly increased migration (141). Concordantly, long non-coding MALAT1 expression was found to enhance cell motility through transcriptional and post-transcriptional regulation of motility related gene expression (142), displaying the strongest association with genes involved in cancer, like cellular growth, movement, proliferation, signaling and immune regulation genes (141). MALAT1 and thymosin β4 expression levels were identified as prognostic parameters for patient survival in stage I NSCLC that are at high risk to develop metastasis (*p* = 0.04 and *p* = 0.01, respectively) (143). Tumorigenesis and metastases may be driven by tumor suppressive and oncogenic pathways deregulation through aberrant expression of cancer metastasis-associated lncRNA (144). In a recent *in vitro* study, the lncRNA brain cytoplasmatic RNA 1 (BCYRN1) was found up-regulated and targeted by c-MYC in human NSCLC cell lines (145). c-MYC is a commonly inhibited oncogene and becomes activated in oncogenic pathways, and correlates with metastasis of NSCLC (146). Besides demonstrating that IncRNA BCYRN1 is essential in the c-MYC-regulated cell migration and invasion, BCYRN1 positively correlates with the expression levels of MMP9 and MMP13 (145). MMP9 and MMP13, two members of the matrixin subfamily of the metzincin superfamily of Zn-dependent metalloproteinases (147), are extracellular matrix degrading proteins proven to induce migration and invasion of tumor cells (147, 148), thereby regulating cancer cell metastasis (149).

## Concluding Remarks

Lung adenocarcinoma establishes distant clinical detectable metastasis within months of initial diagnosis (26, 150). This short abeyance indicates that metastatic ability would arise from early oncogenic events that stimulate primary tumor growth rather than late-arising, scarce genomic alterations specific for metastasis (151). Thus, monitoring persistent chromosomal changes in the primary NSCLC alongside with prospective multicenter studies of patient-matched primary and CNS metastatic lesions could help identify targetable approaches for brain metastasis-specific signatures.

## Author Contributions

RP, DM, JA, and JK wrote and revised the article.

## Conflict of Interest Statement

The authors declare that the research was conducted in the absence of any commercial or financial relationships that could be construed as a potential conflict of interest.
